# Unveiling Candidate Markers for Drug Resistance or Synthetic Lethality in Cervical Cancer: Integrative Analysis of Genetic and Pharmacoprofiling

**DOI:** 10.1002/cnr2.70599

**Published:** 2026-06-12

**Authors:** Suzy Scholl, Elaine Del Nery, Pierre Gestraud, Maral Halladjian, Elodie Girard, Adèle Soria, Elodie Anthony, Leanne De Koning, Nicolas Servant, Els Berns, Maud Kamal

**Affiliations:** ^1^ Department of Drug Development and Innovation (D3i) Institut Curie, Paris‐Saclay University Paris France; ^2^ Biophenics High‐Content Screening Laboratory, PICT‐IBiSA, Paris Biofoundry/UMR168 Department of Translational Research, Institut Curie, PSL Research University Paris France; ^3^ Computational Oncology, PSL Research University, Mines Paris Tech, INSERM U1331 Paris France; ^4^ Department of Translational Research Institut Curie, PSL Research University Paris France; ^5^ Department of Medical Oncology Erasmus MC Rotterdam the Netherlands

**Keywords:** BioRAIDS, cell lines, cervical cancer, epigenetic targeting, genetic biomarkers, HPV, microtubule targeting, pharmacoprofiling, protein biomarkers, standard therapy

## Abstract

**Introduction:**

Systemic cervical cancer management continues to be challenging. Numerous chemotherapies have been approved, but predicting response is difficult due to the lack of biomarkers. Here, we analyze the genetic and protein profiles of 20 cervical cancer cell lines (CCCLs) and explore their correlation with drug response patterns to commonly used drugs, aiming to identify novel biomarkers of treatment response or resistance.

**Material and Methods:**

Twenty cell lines (CLs) were characterized for HPV type, for genetic alterations, and protein expression profiles. Pharmacoprofiling in 10 selected CLs was carried out against 34 drugs used in the clinic, assessing drug concentrations needed to reach half maximal inhibitory concentration (IC50) in nanomolar and micromolar ranges. Subtractive bioinformatics analyses aimed to identify genetic alterations (609 genes of clinical interest), associated with CL drug resistance or on the contrary with synthetic lethality.

**Results:**

Despite a small sample size, genetic alteration frequencies and types of CCCLs were in line with those in clinical samples, except for the detection of a higher frequencyin specific genetic alterations such as *NBPF1* and *STK11* in CLs. Pharmacological screening identified drugs exhibiting therapeutic activity in most CLs while others were highly selective. Bioinformatics analyses suggested, loss‐of‐function (LoF) alterations in *PAPBC3* in CLs sensitive to microtubule interfering agentsin addition to 50 variably present alterations in the microtubule pathway. LoF alterations in *CSMD3, OBSCN, ZNF 717, ALPK2, CLDND1, GTF3A, NLRP1, SI,* and *TRIM66* were associated with Epigenetic acting drug activity and LoF of *OSBPL1A* with Eprenetapopt (APR‐246) activity. Drug synergistic effects were observed with certain drug combinations.

**Conclusion:**

This paper reports genetic variants in 20 CLs as well as the results of the assessment on whether those variants may help predict response or resistance to certain drug families. With a few exceptions, genetic alteration frequency in CCCLs, conducted in the same analytical batches, compares favorably with published patient data. Results need confirmation in independent larger studies both in CLs and in clinical settings.

## 1 Introduction

Six weekly courses of carbotaxol prior to chemoradiation were recently shown to improve patient outcome [1]. However, not all patients benefited from this combined treatment, and predictive biomarkers for response to this combined treatment are lacking. Previous research has already gathered substantial genetic data from well‐documented cervical cancers (CCs) [2, 3, 4].

Cervical cancer cell lines (CCCLs) exhibit similarities with patient tumor samples, often showing resistance to platinum salts, mirroring the behavior of CCCLs typically derived from advanced or refractory disease. CLs differ from tumors by their ability to proliferate in the absence of a tumor microenvironment or a vascular supply. Unsupervised genetic profiling had shown that CCCLs genetic variants cluster with the “DNA‐damage signaling” cluster in primary CCs [5].

The limits of pharmacogenomics, in relation to the necessity of two specific hits for synthetic lethality to a defined drug is well discussed in previous studies [6, 7]. By applying bioinformatic filters to clinically well‐defined genetic alterations or protein expression changes, we screened for biomarkers to discriminate between drug resistance and sensitivity.

The present study builds on this approach by performing pharmacological profiling of patient‐derived cell lines (CLs) alongside comprehensive genetic testing.

## 2 Material and Methods

### 2.1 Cell Culture

In pharmacoprofiling assays we tested drug sensitivity scores (DSS) for clinically active drug families, such as four microtubule targeting agents (MTAs), two epigenetic targeting agents (ETAs), receptor tyrosine kinase inhibitors (RTKIs), metabolic inhibitors, and some novel targeted drugs. Genomic analysis was based on 20 CLs including 16 public CLs, described in Kloth et al. [8] and all CL data are recapitulated in Table 1 [10]. The access of CLs and all details related to the culture conditions are detailed in the Data S1.

**TABLE 1:** Cell line (CL) characteristics.

CL name	Barcode	CL origin	CL hierarchical clustering proximity	Second step of combined pharmacoprofiling	Patient age (at diagnosis)	Pathological diagnosis	Patient lymph node status	CL HPV type	Patient FIGO stage 2014	Patient survival	Ethnicity
SW756	CRL10302	ATCC	NA	Yes	46	SCC	NA	18	NA	NA	Caucasian
CaSki	CRL1550	ATCC	NA	Yes	40	SCC	NA	16	IV	NA	Caucasian
C‐4 I	CRL1594	ATCC	C‐4I and C‐4II	NA	41	SCC	NA	18	NA	NA	Caucasian
C‐4 II	CRL1595	ATCC	C‐4I and C‐4II	NA		SCC	NA	18	NA	NA	Caucasian
Ect1/E6E7	CRL2614	ATCC	Ect1/E6E7 and SiHa	NA	43	SCC	NA	HPV 16 transformed	NA	NA	NA
SiHa	HTB35	ATCC	Ect1/E6E7 and SiHa	NA	55	SCC	NA	18	NA	NA	Asian
DoTc2‐4510	CRL7920	ATCC	NA	Yes	NA	SCC	NA	UKN	NA	NA	NA
C‐33 A	HTB31	ATCC	NA	Yes	66	SCC	NA	NEG	NA	NA	Caucasian
HT‐3	HTB32	ATCC	NA	NA	58	SCC	NA	NEG	NA	NA	Caucasian
ME‐180	HTB33	ATCC	NA	Yes	66	SCC	NA	68	IV	NA	Caucasian
MS 751	HTB34	ATCC	NA	NA	47	SCC	NA	18	III	NA	Caucasian
CSCC‐7	P26	NKI	NA	NA	33	Large cell SCC	Negative	16	IB/IIA	DF > 5 years	Surinam
CC‐11	P49	NKI	NA	Yes	62	ASCC	Positive	67	IIA	4 months	Surinam
CC‐8	P55	NKI	NA	NA	30	ASCC	Positive	45	IIA	16 months	Surinam
CC‐10B	P71	NKI	CC10B and CC10A	NA	45	« glassy »ASCC	Negative	45	IB	DFS > 5 years	Surinam
CC‐10A	P82	NKI	CC10B and CC10A	NA	45	« glassy »ASCC	Negative	45	IB	DFS > 5 years	Surinam
IC1	IC1	Curie	NA	Yes	30	SCC	Positive	18	IB	DFS > 8 years	Caucasian
IC3	IC3	Curie	NA	Yes	26	ASCC	Negative	16	IB		Caucasian
IC4	IC4	Curie	NA	Yes	NA	SCC	Negative	45	NA	< 6 months	Caucasian
IC5	IC5	Curie	NA	Yes	39	SCC	Negative	18	IB	DFS > 10 years	Caucasian

*Note:* Patient FIGO stage 2014 was used since the cell lines were sampled much prior to the present FIGO 2018 which takes into account lymph node staging either by surgical assessment or radiologically. This could not be retrieved with hindsight. Both IB and IIa have no documented parametrial or lymph node invasion. HPV 16 transformed means cells were transfected with HPV [9].

Abbreviations: ASCC, adenosquamous cell carcinoma; ATCC, American tissue culture collection; CL, cell line; DFS, disease free survival; HPV, human papilloma virus; NA, not available; SCC, squamous cell carcinoma.

### 2.2 Screening Workflow

The study investigated several drug families, targeting: DNA repair inhibition (Cisplatin, Carboplatin and Parp inhibition by Olaparib); ETAs: Vorinostat, Azacytidine through histone deacetylation or UNC1999 through EZH2 inhibition; receptor tyrosine kinase inhibition (EGFR pathway/VEGFR/PDGFR); nuclear hormone receptor inhibition; inhibition of energy metabolism (biguanides); antimetabolites (5FU, Methotrexate, Gemcitabine); MTAs: Paclitaxel, Colchicine, Vinblastine, Vinorelbine; proteasome degradation; and p53 modulation (APR‐246).

The clinically approved drugs we tested are listed in Table 2. CLs densities, viability, drug response assessments and the pharmacological compounds that were assessed are further detailed in Data S1.

**TABLE 2:** : List of drugs used.

Name	Ref.	Provider
CH5132799	S2699	Selleckchem
Mk‐2206 2HCl	S1078	Selleckchem
Omipalisib (GSK2126458, GSK458)	S2658	Selleckchem
Erlotinib	S7786	Selleckchem
Gefitinib	S1025	Selleckchem
Lapatinib	S2111	Selleckchem
Imatinib	S2475	Selleckchem
Phenformin HCl	S2542	Selleckchem
Vismodegib (GDC‐0449)	S1082	Selleckchem
Dasatinib	S1021	Selleckchem
Sorafenib	S7397	Selleckchem
Olaparib	S1060	Selleckchem
Vinblastine sulfate	V1377	Sigma
Vinorelbine Tartrate	S4269	Selleckchem
Crizotinib	S1068	Selleckchem
Methotrexate		Pharmacie Curie
Gemcitabin HCl	G6423	Sigma
Metformin HCl	1 396 309	Sigma
RO4929097	S1575	Selleckchem
Azacitidine	S1782	Selleckchem
Vorinostat	S1047	Selleckchem
Bortezomib	S1013	Selleckchem
Colchicin	C9754	Sigma
Carboplatin		Pharmacie Curie
Cisplatin		Pharmacie Curie
UNC1999	S7165	Selleckchem
PFI‐3	S7294	Selleckchem
L6		Marc Billaud
Paclitaxel		Pharmacie Curie
Mitomicyn C		RT^2^ lab
Herceptine		BFX lab
GSK650394	S7209	Selleckchem
APR246		APREA AB
Palbociclib	S1116	Selleckchem

### 2.3 Whole Exome Sequencing (WES)

WES of 20 CCCLs available to the RAIDS consortium was based on Illumina paired‐end sequencing: for 16 public CLs, described in Kloth et al. [8], sequencing was done at SeqOmics, Mórahalom, Hungary while for four CC patient derived CL the sequencing was done at Institut Curie.

### 2.4 Genomic Variant Calling in CCCLs

In the absence of paired germline samples, variant calling of small‐scale alterations (SNVs, InDels) was performed on samples using GATK HaplotypeCaller (v4.0.2.1) using a base quality of 13 for a base to be called. Variants were then annotated using annovar (v 2017/07/06) with respectively gene (refGene), polymorphisms (avsnp v147, 1000G v 2015/08, ESP6500 v2, ExAC) and cancer databases (COSMIC v70, ICGC v21) as well as deleterious impact predictors (dbNSFP v33). Excluding synonymous variants, variants passing the technical filters (GATK best practices), with an allelic ratio higher than 5% and with a depth of coverage above 10× were kept for further analysis. To palliate the lack of matched germline samples, putative germline variants were removed by filtering out polymorphisms with a minor allele frequency (MAF) higher than 1% in either 1000 g, Exome Sequencing Project (ESP) or Exome Aggregation Consortium (ExAC). Copy number alterations were assessed using Facets (v0.5.11), a copy number tool that takes advantage of both the B‐allele frequency (BAF) and the read depth to estimate the allele‐specific copy number variants and the optimal ploidy and tumor cellularity, after a GC percentage correction. Facets needs a control sample to normalize both read depth and BAF profiles and performs well with a matched control. An unmatched control can also be provided if specified by the algorithm. Four different CC patient‐derived blood samples from the BioRAIDs population were tested to generate the copy number profiles for the 20 CLs: these were patient numbers 101‐002, 101‐008, 101‐032, 102‐012. A correlation heatmap (Pearson test) (Figure S5) on log ratio profiles was generated to highlight a putative better control sample. The 101‐002 sample was considered as control sample for further analysis. Based on the decision algorithm, only nonsynonymous SNVs predicted as deleterious by Sift & Polyphen or nonsynonymous presenting a COSMIC id were kept for all 3 classes of genes: Oncogenes (O), Tumor‐Suppressor Genes (TSGs) as well as Genes of Presently Unproven Significance (GPUS). For oncogenes: frameshift INDELs; for tumor‐suppressor and genes of unknown significance the following: stop‐gain, frameshift INDELs, and splice variants were considered. To qualify for TSGs, only nonsynonymous SNVs present in a Loss of Heterozygocity (LOH) locus were kept (double hit). Altered genes had been annotated, based initially on the BioRAIDs patient panel. Mutations of known significance in patients, provided by the ATCC database were used as validations points. Among the 4915 additional variants (3872 distinct variants), 588 were called in more than 20% of the CLs, in the “of unknown significance” gene panel (Data S1 and Table S1).

### 2.5 Reverse Phase Protein Arrays (RPPA)

Twenty CLs had been processed together with the 154 baseline tumor samples and 103 posttreatment samples as previously reported [2]. Arrays were labeled with 194 specific antibodies (Table S2) using an Autostainer Plus (AGILENT). All primary antibodies used in RPPA had been previously tested by Western Blotting to assess their specificity for the protein or phosphoprotein of interest [2, 3, 5].

### 2.6 Bioinformatics Analysis of Genetic Alterations Potentially Linked With Drug Response

Genetic alterations potentially linked with drug response were assessed, focusing on genes altered in at least two CLs with available drug response data, or genes altered (gain or loss of function) in all good or bad responders. A broader screen, including genetic variants of presently unproven clinical significance (Figure S1) was employed to identify markers associated with therapy resistance or synthetic lethality.

## 3 Results

### 3.1 Genomic Profiling of CCCLs

Tocorrelate drug response with molecular characteristics of CC, We assembled a panel of 20 CLs, including 16 public CL (described in Kloth et al. [8]) and four in‐house CLs available at Curie Institute [10]. First, we established the genomic profiles of these CCCLs. Gene annotation was based on the previously described RAIDs panel in primary tumors. It was initially restricted to 100 oncogenes, 203 TSGs used routinely for patient evaluation, and 306 gene alterations of potential clinical significance, but was expanded later to the whole exome to include genes not typically considered by clinical tumor boards.

**FIGURE 1:**
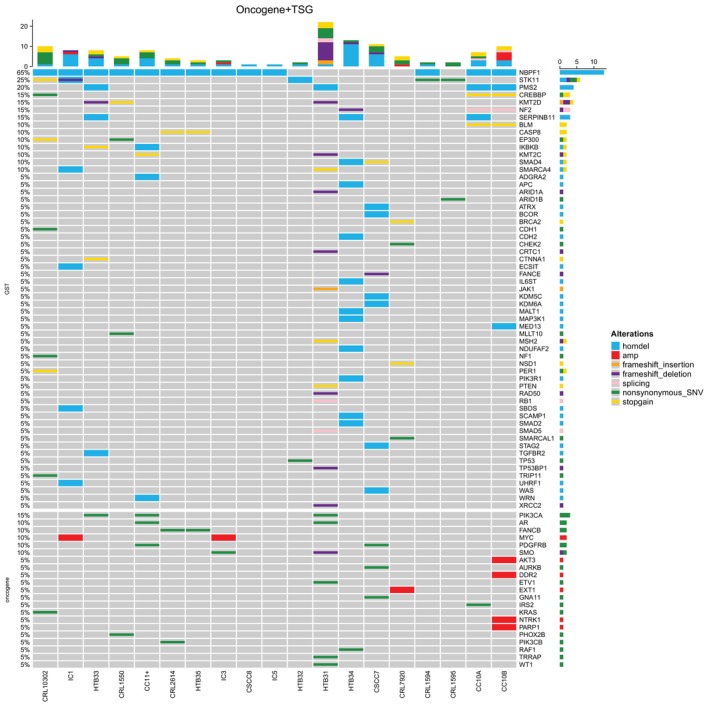
Oncoprint: Matrix based visualization that summarizes genomic alterations enabling rapid assessment of mutation frequency and type of alteration for each of the 20 CLs together with probable function. amp, amplification; Homdel, homozygous deletion; O, oncogene, SNV, single nucleotide variant; TSG, tumor suppressor gene.

The frequency and types of genetic alterations for each CL are illustrated in “Oncoprint” (Figure 1 and Table S1).

Hierarchical clustering based on polymorphisms highlighted clustering of CRL1594 (C4‐I) with CRL1595 (C4‐II), as well as of CC10A (passage 72) with CC10B (passage 81 of the same patient) as expected, but it also showed a proximity between Ect1/E6E7 (CRL2614) and SiHa (HTB35) CLs, which had not been previously described (Figure S2). We therefore considered these CLs as duplicates and excluded them from further pharmacological profiling. The rate of clinically significant genetic alterations detected in CLs was comparable to the clinical samples with two notable exceptions. Homogeneous deletions (HOMDEL) leading to loss‐of‐function (LoF) of Neuroblastoma Breakpoint Family Member1 (*NBPF1*) were present at significantly higher frequencies in CLs (65%) than in primary tumors (BioRAIDs: < 1%). LoF gene alterations (by either HOMDEL, stop‐gain, or nonsynonymous SNV) *of STK11* (Serine/Threonine Kinase 11), a Polarization‐Related Protein, were present in 5/17 (29%) as compared to 7% in the BioRAIDs population (final paper submitted). In epigenetic‐modifier genes the following LoF alterations were seen: *KMT2D* in 3/17, *CREBBP* in 3/17, while *ARID1A*, *ARID1B, TP53*, *PTEN* LoF were detected in 1 CL each. IC1 and IC3 were noteworthy for a c‐*MYC* gene amplification. Activating mutations in *PIK3CA* were present in 3/17 CLs. CC10B was different to its sister CL (CC10A) by a gene amplification burst with additional genetic alterations (including ABL2, AKT3). Oncogenic alterations in *PIK3CB* were found in Ect1/E6E7 (CRL2614), while DoTc2‐4510 (CRL 7920) had a nonsynonymous alteration in *BRCA2*.

### 3.2 Micro‐Satellite Instability

In a SNV/INDEL ratio analysis, only HTB31 had a significantly higher number of frameshift deletions and noncoding variants, with > 18 000 INDELs in a raw analysis, while the average number across CLs was 7000–8000. Postfiltering, INDELs of significance averaged 20–40 per CL, with HTB31 close to 500, while SNV averaged 500–1000 by CL, with HB31 being out of range with 1900 SNV. MSI‐sensor verification classified HTB31 as MSI‐High (Table S3).

Human papillomaviruses (HPVs), responsible for over 90% of CCs, were detected in 17/19 (89%) of CLs (Table 1) meaning that the rate of HPV infection was similar to that of clinical samples.

### 3.3 RPPA Data on Cell Lines and Drug Response

Next, we characterized the CCCLs for their expression profiles of 194 proteins and phosphor‐proteins using RRPA. Three main RPPA clusters with different protein expression profiles were identified in patient samples. Pathway enrichment analysis highlighted DNA damage signaling (cluster 2), MAPK/PI3K signaling (cluster 1), and EMT and ErbB signaling in CLs. All CLs had low CD45 expression, consistent with the absence of microenvironmental cells.

### 3.4 Drug Sensitivity Scores and Heatmap of Drug Activities

**FIGURE 2:**
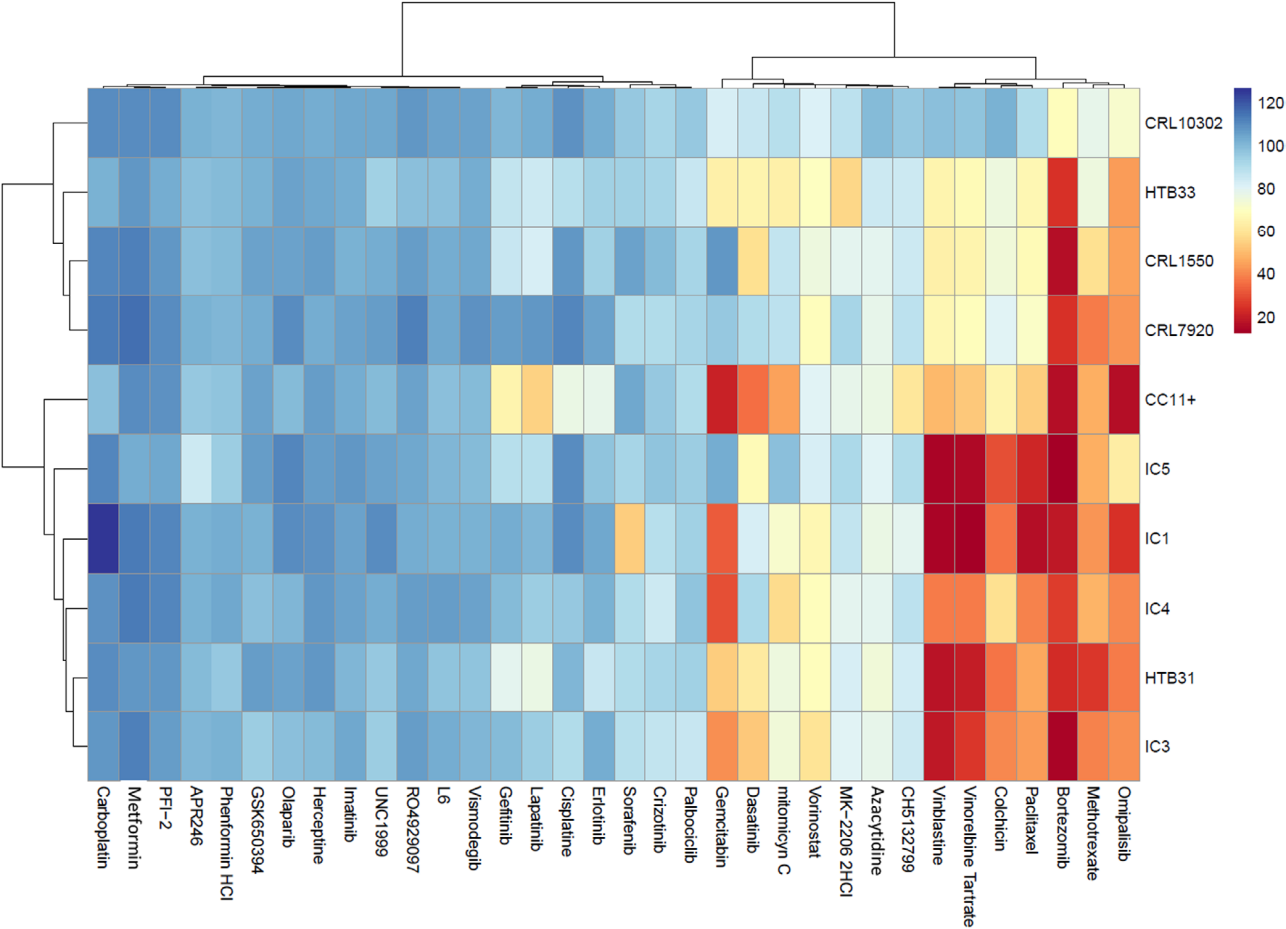
Heatmap—pharmacoprofiling cell viability response profile clustering reveals differential drug‐sensitivities across cervical cancer cell models with distribution of DSS response at the compound level from highly responsive (red) and lower sensitivity (blue). Bortezomib, Methotrexate, Omipalisib, Azacytidine, Vorinostat, and Gemcitabine showed significant activity in virtually all cell lines. All microtubule targeting reagents: Paclitaxel, Colchicine, Vinorelbine and Vinoblastine showed activity in the same cell lines. Cisplatin and Carboplatin showed no significant activity.

A pharmacological screen using 34 molecules (Table 2) known to interfere with specific signaling circuitries or clinically established cancer drugs was conducted in 10 selected CLs. DSS considered both the slope of the curves and the drug concentrations needed to reach IC50 (Figure S3). Response profiles were assessed for their spectrum of activity (broad or selective) as well as for the drug concentrations needed to reach IC50, as visualized in the heatmap (Figure 2) from the nanomolar (nM‐red) to the 10 micromolar (10uM‐blue) range. Bortezomib, Omipalisib, and ETA class (epigenetic targeting) agents showed broad‐spectrum activity while drugs in MTA class (microtubule targeting) agents as well as Eprenetapopt (APR‐246), Gemcitabine, Methotrexate, and Sorafinib showed activity only in selected CLs.

1. Significant DSS of drugs active in the nanomolar range
  - Drugs with a broad activity spectrum ≥ 8/10 CLs

Bortezomib, a proteasome inhibitor, exhibited remarkable sensitivity in all 10 CLs. Methotrexate, an antimetabolite targeting the DHFR enzyme, showed medium to high DSS in 8/10 CLs at a concentration of 50 nM. Of note: most CLs had above median di‐hydro‐folate reductase (DHFR) values, the overexpression of which has been linked to methotrexate resistance. Methotrexate works by inhibiting DHFR, an enzyme crucial for the synthesis of tetrahydrofolate, a necessary building block for replication.

b Drugs with an intermediate activity spectrum: 3–6 CLs

Three CLs (IC1, IC3, IC5) had the same spectrum of activity to all four MTAs tested (Paclitaxel, Colchicine, Vinblastine, Vinorelbine), while SW756 (CRL 10302) was resistant to all four reagents, and DoTc2‐4510 (CRL 7920) and Caski (CRL 1550) were sensitive in the micromolar range only, suggesting pharmacological activity is independent of the specific reagent or the supposed mode of action by stabilization or destabilization of microtubules.

Gemcitabine, an analog of deoxycytidine which inhibits DNA synthesis, revealed high activity at 5–10 nM concentrations in IC1, IC4, and CC11; intermediate activity in C‐33 A (HTB31) and ME‐180 (HTB33); and low activity in IC5, in SW756 (CRL 10302), DoTc2‐4510 (CRL 7920), and Caski (CRL 1550).

c Drugs with a selective activity

EGFR pathway inhibition was effective in CC‐11, which showed sensitivity to CH5132799 and Omipalisib.

Sorafenib exhibited selective activity in IC1 at 0.1 uM concentrations, while other CLs required significantly higher concentrations (> 10 uM).

2 Significant DSS of drugs active in the micro‐molar range (5–10 uM)

- Drugs with a broad activity spectrum: ≥ 8/10 CLs.

Omipalisib, a PI3K/MTOR inhibitor, showed high DSS in 8/10 CLs, despite that PIK3CA activating mutations were detected in only 3/10 CLs.

For drugs in the class of ETAs, HDAC inhibition by Vorinostat or Azacytidine was highly effective at 5 uM concentration in eight CLs, with a lag in response in IC5 and SW 765 (CRL 10302). UNC 1999, an orally bioavailable chemical probe of the Lysine‐Methyltransferases EZH2 and EZH1 (enhancer‐of‐zeste‐homolog‐1 and 2) [10, 11, 12, 13] had a similar activity spectrum in 7/10 CLs, with a lag in response for three CLs: IC5, SW756 (CRL 10302) and Caski (CRL 1550), responding at 10 uM concentrations.

Palbociclib (DK4/CDK6 inhibitor acting via Rb) and Crizotinib (inhibitor of receptor tyrosine kinases) had significant DSS across all CLs. MK‐2206 [14], an allosteric AKT inhibitor able to suppress the Akt phosphorylation induced by carboplatin and gemcitabine [15]—with demonstrated activity in the clinic—was active in all CLs.

b Selective drug activity

The p53 “modulator” APR‐246 (Eprenetapopt) was effective in three CLs: IC3, IC5, and CC‐33A (HTB31) (Figure S3).

3 Reagents without significant activity in any CL

Platinum exhibited minor response in CLs, as expected in cells from patients whose tumors had evaded treatment with platinum salts. CC11 showed the best response to Cisplatin, Carboplatin, and Olaparib, while the remaining CLs appeared resistant. There was partial concordance in protein expression (RPPA) patterns between platinum‐resistant CLs and treatment‐resistant patient samples. Of note are the above‐median expression levels of the following activated proteins: HSF1, PKM2, NBS1, and EGFR, which had been associated with poor outcome in patients treated by chemoradiation (by CoxBoost analysis), and which are present in virtually all CLs. Similarly, expression of IDO, p27, and PTP1B, which had been associated with treatment response in patients, was mostly downregulated in CLs.

None of the CLs tested showed any activity to the following: Imatinib (stabilizer of the inactive, non‐ATP binding form of BCR/ABL), Metformin, Phenformin (in vivo action on glucose metabolism and in vitro action on mitochondrial respiration), PFI‐2 (potent and selective inhibitor of the methyltransferase activity of SETD7), RO4929097 (gamma secretase inhibitor), Herceptin (consistent with absence of HER2 gene amplification), or GSK650394, a novel SGK (serum and glucocorticoid regulated kinase) inhibitor. In terms of protein expression (RPPA), the enzyme PFKFB2, a potent activator of glycolysis, had a significantly higher expression level in metformin resistant CLs, as previously published [5].

4 Highly resistant or highly responsive CLs

SW756 (CRL 10302) and DoTc2‐4510 (CRL 7920) were highly resistant to most drugs.

IC1 and CC‐11 demonstrated significant drug sensitivity, including Sorafenib targeting the RAF/MEK/ERK pathway and RTK inhibition, all four MTAs, three ETAs, Methotrexate, Gemcitabine, Bortezomib, and Palbociclib (CDK4/CDK6 inhibitors via Rb). CC‐11, but not IC1, also showed sensitivity to Dasatinib, Platinum, Olaparib, Mitomycin C, and EGFR inhibitors.

5 Synergy: Bliss Index

**FIGURE 3:**
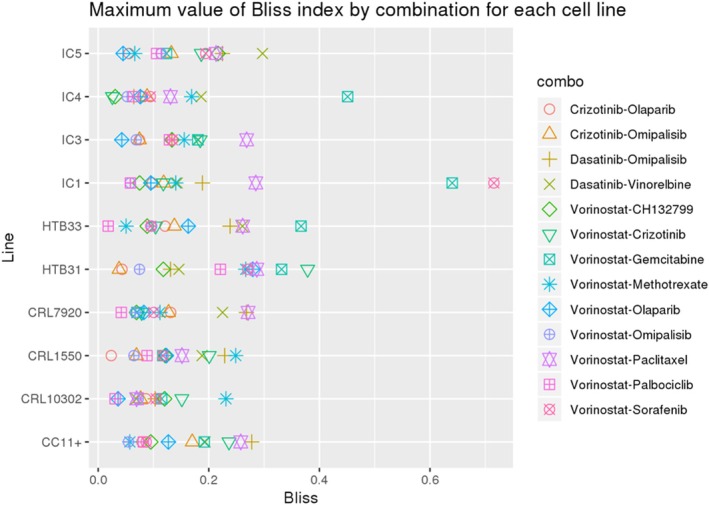
Bliss Index as test for drug interactions, showing the summary for each drug combination by cell line. The Bliss index quantifies drug–drug interactions by comparing the observed combined effect to the expected effect under a model of independent action, enabling classification of combinations as synergistic, additive, or antagonistic. Synergistic effects were observed using the combination (Combo) Vorinostat‐Methotrexate for highly resistant CLs: SW756 (CRL 10302) and Caski (CRL 1550) and the combo Dasatinib with Vinorelbine or Omipalisib for DoTc2‐4510 (CRL 7920). Vorinostat‐Gemcitabine showed synergistic activity in IC1 and IC4. Vorinostat‐Sorafenib showed synergistic activity in IC1.

Synergistic effects were observed with certain drug combinations in specific CLs, such as Vorinostat and Methotrexate for the highly resistant CLs to most drugs: SW756 (CRL 10302) and Caski (CRL 1550) (Figure 1), and Dasatinib with Vinorelbine or Omipalisib for DoTc2‐4510 (CRL 7920). Vorinostat with Gemcitabine showed synergistic activity in IC1 and IC4 and Vorinostat with Sorafenib showed synergistic activity in IC1 (Figure 3 and Figure S4).

### 3.5 Genomic Correlates for Drug Resistance (Table 3)

**TABLE 3:** Genes extracted in relation to response/resistance by drug family.

Genes extracted	Biological system targeted	Gene alteration (GA) detected incidence in patient tumors (*n* = 301)
*ZNF717*	Nondefined	7 (2.3%)
*HYDIN*	Nondefined	8 (2.7%)
*CTBP2*	Nondefined	3 (1.0%)
*CTDSP2*	Nondefined	NA
*FAM104B*	Nondefined	NA
*PABPC3*	Microtubules	5 (1.7%)
*CSMD3*	Epigenetic	33 (11%)
*OBSCN*	Epigenetic	26 (8.6%)
*ZNF 717*	Epigenetic	7 (2.3%)
*ALPK2*	Epigenetic	4 (1.3%)
*CLDND1*	Epigenetic	1 (0.33%)
*GTF3A*	Epigenetic	0 (0%)
*NLRP1*	Epigenetic	1 (0.33%)
*SI*	Epigenetic	9 (3%)
*TRIM66*	Epigenetic	NA
*OSBPL1A*	Metabolism	3 (3.0%)

The “Bio‐RAIDS” gene‐set was utilized for bioinformatics subtraction analysis to identify genomic correlates that could differentiate between sensitive and resistant CLs. The initial gene list was expanded to include genes not typically considered by clinical tumor boards. The results are considered to be exploratory due to the limited number of CLs undergoing pharmacoprofiling and compared to the vast number of alterations discovered.

1. Genomic correlates for resistance to any drug treatment

Subtractive computational analysis identified LoF alterations in *CTBP2, CTDSP2, FAM104B, HYDIN*, and *ZNF717* as potential genetic markers for drug resistance. These alterations were consistently present in at least two CLs resistant to any drug but absent in all good responders. Frequencies of *HYDIN* and *ZNF717* alterations in CLs were approximately 7%, while their presence in primary patient samples is low (1%–2%) (Table 3).

2 Markers of sensititvity or resistance to MTAs: Paclitaxel, Vinorelbine, Vinblastine, and Colchicine

MTAs are well‐known drugs that disrupt the mitotic spindle, halt the cell cycle at M phase, and cause apoptosis. MTA‐sensitive CLs showed consistent responses to all four MTA family members (Figure 2). Through bioinformatics subtraction, a LoF alteration in *PABPC3* (Poly A tail binding protein, cytoplasmic C3) was identified in a nonresponder and absent in responders, while an additional 50 other gene alterations in the microtubule pathway were variably present. *PABPC3* is involved in RNA stability and translational initiation. LoF alterations were present in 10/301 (0.3%) patients from the RAIDS database, with five so far being validated by Cosmic, ICGC, or Cancer Hotspot databases. BioRAIDS patients had not been exposed to drugs from the MTA group.

3 Genomic correlates for resistance to ETAs

Eight of 10 CLs showed intermediate to high sensitivity to HDACs (Vorinostat, Azacytidine), aligning with LoF alterations in epigenetic modifier enzyme genes. Seven of these CLs also responded equally to UNC 1999, an EZH2 and EZH1 inhibitor. Specific LoF genetic alterations in *CSMD3, OBSCN, ZNF 717, ALPK2, CLDND1, GTF3A, NLRP1, SI*, and *TRIM66* were found in two resistant CLs but not in the seven sensitive ones. In patients from the BioRAIDS dataset, any *CSMD3, OBSCN*, and *SI* alterations were seen in 26%, 25%, and 10% of cases.

4 Genomic correlates for synthetic lethality by APR‐246 (Eprenetapopt)

*OSBPL1A*, part of the oxysterol‐binding protein (OSBP) family, showed LoF alterations in three sensitive CLs but not in the seven resistant ones to Eprenetapopt. This suggests that Eprenetapopt may be synthetically lethal in cancer cells with an *OSBPL1A* LoF. In BioRAIDS patients, *OSBPL1A* alterations were seen in 3% of cases, so far 1% have been validated by official databases such as Cosmic or ICGC.

These findings provide insights into potential genetic markers associated with drug resistance across different classes of drugs, aiding in the understanding of treatment response or resistance mechanisms.

## 4 Discussion

Since a linkage between drug activity and specific genetic alterations may reveal new treatment options. We conducted this study combining pharmacological and genetic profiling in CLs to detect key genomic alterations and protein expression changes that could inform on resistance or synthetic lethality to certain drug classes.

1. Bioinformatics extraction for drug resistance markers

- Platinum resistance

Pharmacoprofiling (Figure 2) had highlighted several drugs with broad activity in “platinum‐resistant” CLs, including Bortezomib, Omipalisib and ETA, while MTA, Eprenetapopt, Methotrexate, and Sorafinib showed activity only in selected CLs.

Huang et al. [16] compiled a platinum resistance database (http://ptrc‐ddr.cptac‐data‐view.org) of over 900 genes/proteins associated with platinum resistance. Focusing on genes of potential clinical relevance, our analysis identified LoF alterations in suppressor genes present in at least two resistant CLs but absent in sensitive ones: *ZNF717, HYDIN, CTBP2, CTDSP2*, and *FAM104B*. In the BioRAIDS database, *ZNF717* and *HYDIN* LoF genetic variants were observed in 7% of patients. Validation for clinical relevance of these genetic alterations by databases like Cosmic or ICGC is presently low (1%–2%), but there are literature reports, suggesting their potential relevance. LoF of *ZNF717* has been linked to poor outcome in CC [17], while alterations in *HYDIN* have been associated with recurrence in early‐stage lung cancer [18, 19]. Clinical evidence links *CTDSP2* alterations to resistance to chemoradiation in rectal cancers [20], while *CTBP2*'s involvement in chromatin regulation suggests potential epigenetic roles. Although information on *FAM104B* gene alterations in cancer is limited, the detected variants in this study align with other findings, indicating their potential significance [21].

b Microtubules: MTA resistance modeling

Analysis of four MTAs—Paclitaxel, Vinorelbine, Vinblastine, and Colchicine—revealed uniform effects across all tested CLs, indicating potential common genetic factors influencing drug sensitivity. Six weekly courses of carbotaxol prior to chemoradiation were recently shown (INTERLACE TRIAL—Mc Cormack et al.) to improve patient outcome [1] Bioinformatics filtering in CLs identified LoF alterations in the *PABPC3* gene present in one CL resistant to MTAs, while absent in four CLs showing high sensitivity to this drug family. *PABPC3* is implicated in cytoplasmic regulatory processes, mRNA metabolism, stability, and translation initiation. Although *PABPC3* LoF was found in a resistant CL, additional (> 50) gene alterations associated with the “post‐translational modification of tubulin” pathway varied among resistant CLs and were not uniformly present. In ovarian cancer, mutations in *PABPC1, PABPC3*, and *TFAM* have been linked to treatment resistance and overall survival (OS) [22]. Similarly, in a study of the DNA methylome in visceral adipose tissue, *PABPC3* was identified as biologically relevant for colorectal cancer development [23].

c Epigenetics

ETA resistance or synthetic lethality: Pharmacoprofiling with Vorinostat, Azacytidine, or UNC1999 distinguished nine LoF genetic alterations between ETA‐resistant and ETA‐sensitive CLs: *CSMD3, OBSCN, ZNF 717, ALPK2, CLDND1, GTF3A, NLRP1, SI*, and *TRIM66*. ETAs were previously considered synthetically lethal in certain cancer cells, but only a subset of sensitive CLs showed relevant alterations. *CSMD3* LoF, a prevalent marker for poor outcome postchemoradiation [3, 24], was frequent and clinically validated. *OBSCN* LoF had been linked to response to radiation in esophageal cancer [25]. *ZNF717* LoF, involved in various cellular functions, was reported in association with radiation or chemo‐radiation resistance [25]. Other LoF alterations: *ALPK2, CLDND1, GTF3A, NLRP1, SI*, and *TRIM66* showed associations with different cellular functions or diseases, indicating potential relevance to cancer treatment response or resistance.

d P53 remodeling or ferroptosis

Eprenetapopt's mechanism had been initially thought to involve P53 remodeling. The drug was subsequently considered synthetically lethal for *ARID1A*‐deficient cells, independent of *p53* function. Our data suggest that *OSBPL1A* LoF may be a marker of Eprenetapopt activity, but the connection remains unclear. *OSBPL1A* is involved in the adaptive immune system and lipid metabolism pathways as a sterol binding protein [26]. Diseases associated with *OSBPL1A* alterations include “Splenic Manifestation Of Leukemia” [27]. It has been suggested that Eprenetapopt‐induced cell death can be suppressed by iron chelators and lipophilic antioxidants, suggesting ferroptosis as a mechanism of action.

2 Comparative frequencies of genetic alterations or protein levels in CLs and patient samples

Most clinically detected genetic LoF or oncogenic alterations are observed in CLs. Additional high‐frequency LoF gene alterations detected in the present work included *NBPF1* (65%) and *STK11* (25%). *NBPF1* LoF alterations have been reported in various cancer types, with HOMDELs leading to aberrant protein expression. It was documented to function as a tumor suppressor, as shown by its forced expression in HEK293T cells [28].

While *NBPF1* alteration*s* were rare in primary cancers (< 1%), *STK11* LoF alterations were detected in 7% of European patient samples (BioRAIDS), low as compared to the 16% reported in a study on Japanese CC patients [29]. *STK11*, a known tumor suppressor, regulates cell polarity, energy metabolism, and DNA damage response and has been associated with Peutz‐Jeghers syndrome and prognosis in several cancers, including CC [29, 30].

Protein expression changes in patient tumors, notably HSF1, PKM2, NBS1, and EGFR isoforms, which we have previously shown to correlate with poor outcome (BioRAIDS) [2, 3] were also detected in CLs with low DSS. RPPA data from CLs had been established in the same batch as clinical patient samples but, not surprisingly, not all protein changes associated with progression free survival in patients correlated with drug sensitivity in CLs.

3 Clinical prospects from CC pharmacoprofiling in the context of the literature

Several drugs showed activity across CLs: these were Bortezomib, Palbociclib, Crizotinib, and Omipalisib, at dosages ranging from nanomolar to micromolar concentrations. Bortezomib is reported to induce apoptosis in rapidly dividing cells and is now part of standard first line therapy for myeloma patients [31]. Palbociclib exhibited efficacy in all CLs tested, suggesting it may be of clinical interest in standard treatment resistant tumors. Crizotinib, theoretically targeting c‐Met/ALK, demonstrated activity in CC cells independent of c‐Met/ALK alterations [32]. A response to Omipalisib in most CLs, not linked to *PIK3CA* variants (present in only three CLs), suggests that additional markers may be relevant. Eprenetapopt showed activity in *OSBPL1A* deficient, platinum resistant CC cells, of interest in the context of its clinical use in myelodysplastic syndromes and in oligoblastic AML patients with *TP53* mutations [33]. Recently *OSBPL1A* deficiency was shown involved in lipid metabolism and ferroptosis [26]. Ferroptosis shares some characteristics with cell death. *OSBPL1A* deficiency and a mechanistic relation in the response to Eprenetapopt needs further documentation, in particular since the BioRAIDs study linked tumor necrosis (assessed by an independent pathology review) at baseline to poor outcome in patients in multivariate analyses.

In highly resistant CLs, we saw synergistic effects using the following dual drug combinations: Methotrexate‐Vorinostat, Dasatinib‐Vinorelbine or Dasatinib‐Omipalisib. Due to the small CL sample and the combined drugs approach no mechanistic study was attempted.

Presently, the identification of validated genomic markers for drug sensitivity or resistance remains a challenge. LoF genetic alterations in *CSMD3, ZNF717, ARID1A, CREBBP, TP53*, or activating mutations in *PIK3CA* have been associated with poor responses to standard platinum‐containing chemoradiation therapy in CC [2, 3, 24], which is in line with our data. In the future, samples from clinical trials, which demonstrated clinical benefit in the active arm by the addition of a single drug in a randomized population, such as taxanes (MTA) to standard chemoradiation [1] might be further tested to validate some of our findings and to detect genetic markers linked to sensitivity or resistance to taxanes. Ongoing clinical trials explore combinations of immune checkpoint inhibitors (ICIs) with drugs like Vorinostat, showing potential for enhancing therapeutic efficacy [34]. Considering the presence of HPV in over 90% of CC cases, targeting the virus alongside immune targeting drugs holds significant promise.

## 5 Conclusion

While pharmacoprofiling together with screening for genomic variants may offer valuable insights into drug efficacy as a function of the genetic context and has the potential to detect genetic markers of resistance or added sensitivity to distinct drug classes [6, 7] there are limitations of a direct application of CL studies to the clinic. Screening for drug activity as a function of the tumor genetics in clinical trials (in patients) is not cost effective due to the cost of WES in all patients, the high variability of genetic alterations between patients, the multitude of potential markers in the same pathway as well as the outgrowth of different clones with new genetic markers over time in an individual patient. Additional preclinical work in association with the analysis of samples from past and future clinical trials may help to confirm the relevance of our findings.

While circulating tumor HPV is already used in patient management to monitor residual tumor activity, exploring HPV‐targeted therapies in conjunction with ICIs and or one of the drugs: (Bortezomib, Palbociclib, Crizotinib, Omipalisib) together with exploring genetic resistance/sensibility markers to conventional therapies should prove beneficial to future patient management.

## Author Contributions

**Els Berns:** investigation, writing – review and editing, validation, supervision, resources. **Elodie Girard:** software, formal analysis, data curation, investigation, writing – review and editing. **Pierre Gestraud:** software, formal analysis, methodology, writing – review and editing, investigation, data curation. **Leanne De Koning:** investigation, methodology, validation, formal analysis, data curation, resources, writing – review and editing. **Elaine Del Nery:** investigation, writing – review and editing, methodology, validation, formal analysis, data curation, resources. **Maud Kamal:** conceptualization, investigation, writing – review and editing, methodology, writing – original draft, validation, visualization, funding acquisition, formal analysis, project administration, data curation, supervision, resources. **Suzy Scholl:** conceptualization, investigation, funding acquisition, writing – original draft, methodology, validation, visualization, writing – review and editing, formal analysis, data curation, supervision, resources. **Maral Halladjian:** writing – review and editing, project administration. **Elodie Anthony:** formal analysis, data curation, resources, investigation, writing – review and editing. **Nicolas Servant:** software, formal analysis, methodology, validation, investigation, writing – review and editing, data curation, supervision. **Adèle Soria:** methodology, formal analysis, data curation, resources, investigation, writing – review and editing.

## Funding

This work was supported by the European Unions Seventh Program for Research, Technological Development and Demonstration (304810) and the Fondation ARC Pour la Recherche Contre le Cancer.

## Ethics Statement

Ethical approval has been obtained for the clinical study BioRAIDS to which we refer to in the discussion when comparing frequency of gene alterations. BioRAIDS is published and mentions the ethical approval.

## Conflicts of Interest

The authors declare no conflicts of interest.

## Supporting information


**Figure S1:** Genes of unproven clinical significance in the 20 cell lines. SNV, single nucleotide variant.

**Figure S2:** Hierarchical clustering of the cell lines based on polymorphisms highlighted clustering. IC50, half maximal inhibitory concentration.

**Figure S3:** Drug response profiles (IC50) in 10 selected cell lines. IC50, half maximal inhibitory concentration for drugs tested from nM (1) to 10 μM (10000) ranges. (A) Response profiles of APR246, Azacytidine, Botertomib, Carboplatin, CH5132799, Cisplatin, Colchicin, Crizotinib, Dasatinib, Erlotinib, Gefitinib, Gemcitabin. (B) Response profiles of GSK650394, Herceptin, Imatinib, L6, Lapatinib, Merformin, Methotrexate, Mitomycin C, MK‐2206 2HCI, Olaparib, Omipalisib, Paclitaxel. (C) Reponse profiles of Palbociclib, PFI‐2, Phenformin HCI, RO4929097, Sorafenib, UNC1999, Vinblastin, Vinorelbin tartrate, Vismodegib, Vorinostat.

**Figure S4:** BLISS INDEX summary for each cell line by drug combination. See details under Figure 3. Combo, combination.

**Figure S5:** A correlation heatmap (Pearson test); Pearson's correlation coefficient (*r*) was used to assess the strength and direction of linear relationships between continuous variables, with statistical significance set at *p* < 0.05.

**Data S1:** cnr270599‐sup‐0006‐Supinfo.docx.

**Table S1:** Detailed report of genetic variants.

**Table S2:** List of antibodies used for RPPA. UKN, unknown.

**Table S3:** Microsatellite number of mutations in the 20 cell lines.

## Data Availability

The data that support the findings of this study are available from the corresponding author upon reasonable request.
