# Supplementary figures and images for: Unveiling Candidate Markers for Drug Resistance or Synthetic Lethality in Cervical Cancer: Integrative Analysis of Genetic and Pharmacoprofiling
Source: Cancer Rep (Hoboken). 2026 Jun 12;9(6):e70599. doi: 10.1002/cnr2.70599 (PMC13263414; doi:10.1002/cnr2.70599)

## Slide 1
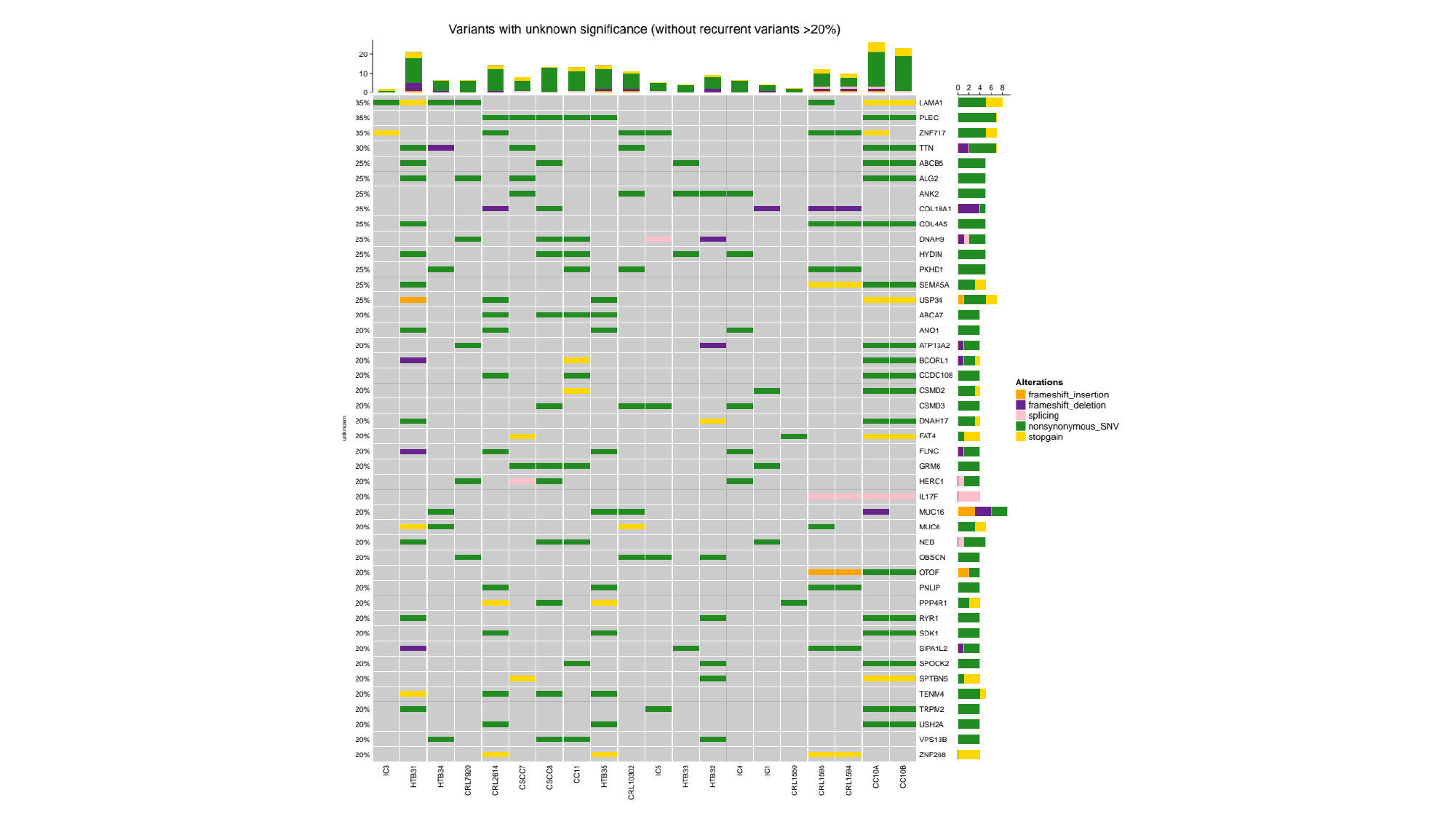

Supplement: Supplementary file 1 — Figure S1: Genes of unproven clinical significance in the 20 cell lines. SNV, single nucleotide variant. [file CNR2-9-e70599-s009.pptx]

## Slide 1
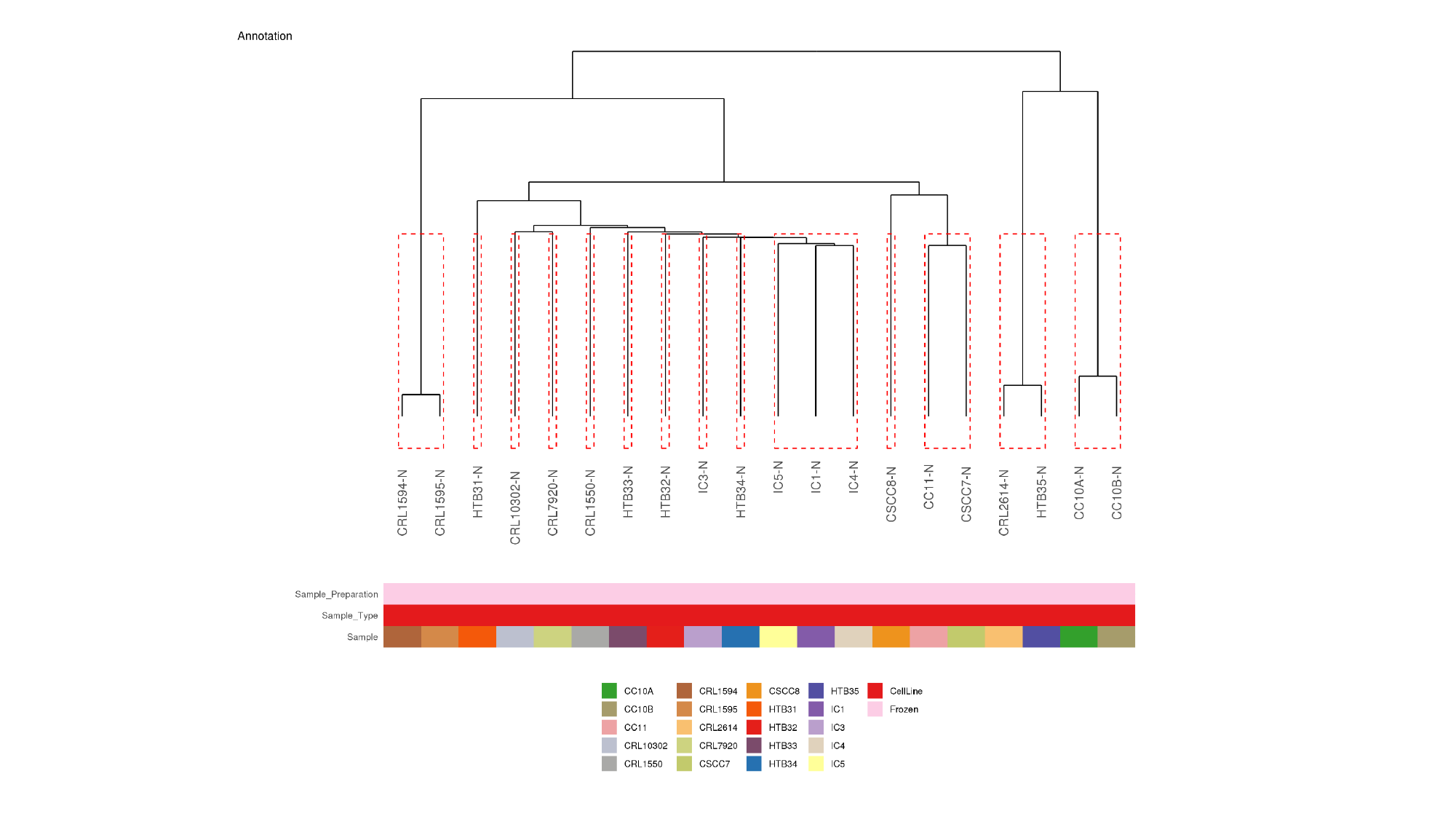

Supplement: Supplementary file 2 — Figure S2: Hierarchical clustering of the cell lines based on polymorphisms highlighted clustering. IC50, half maximal inhibitory concentration. [file CNR2-9-e70599-s001.pptx]

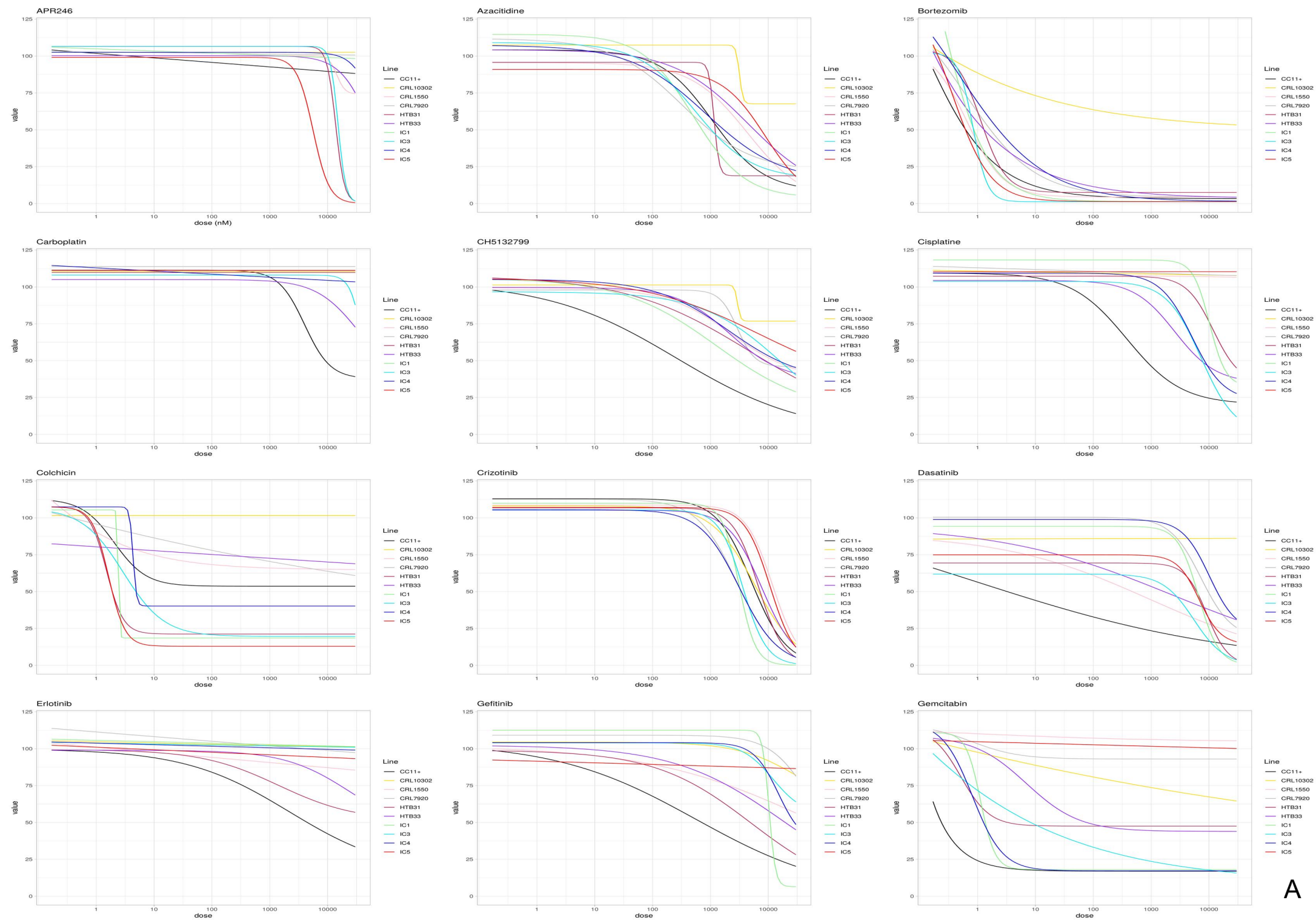

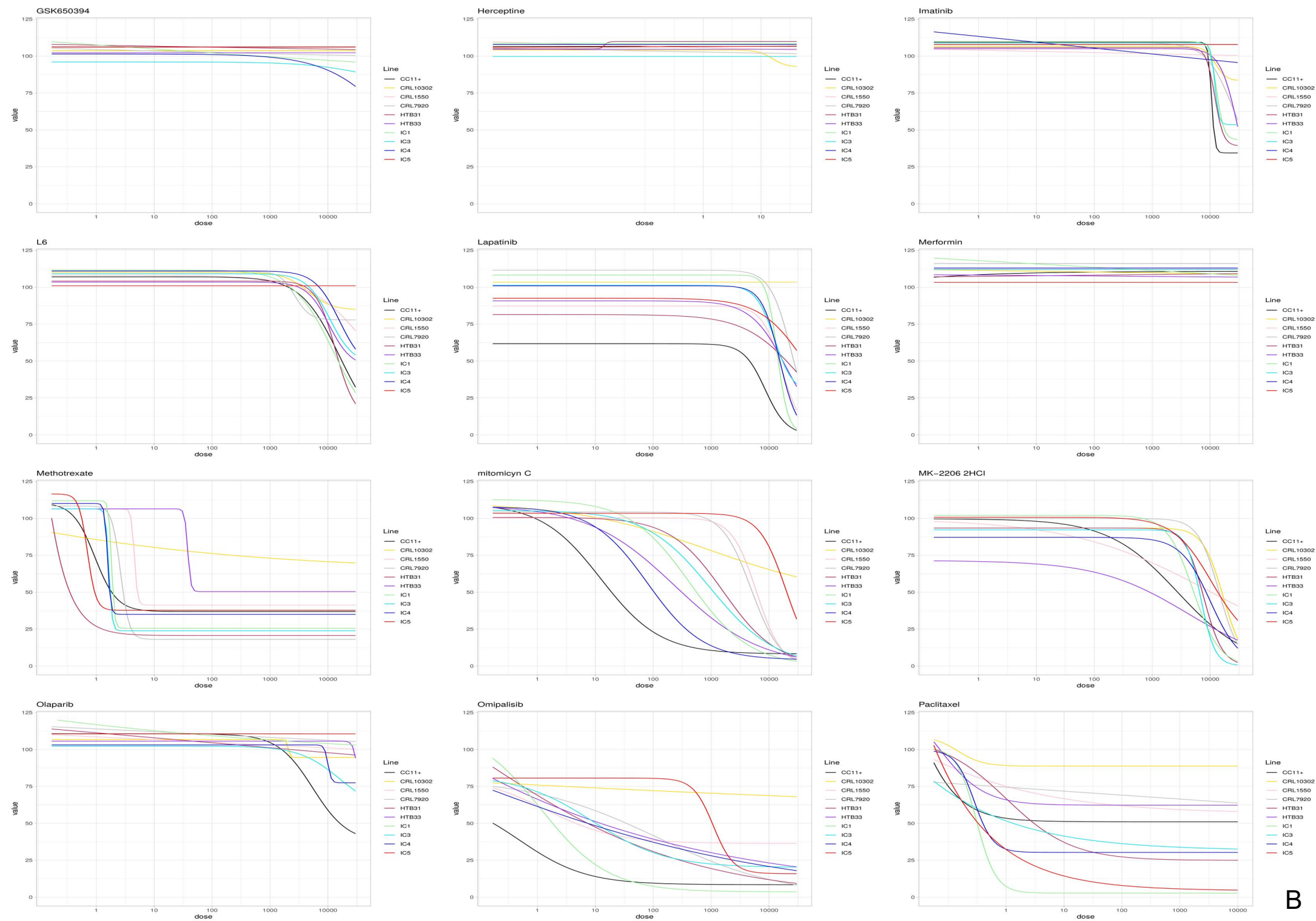

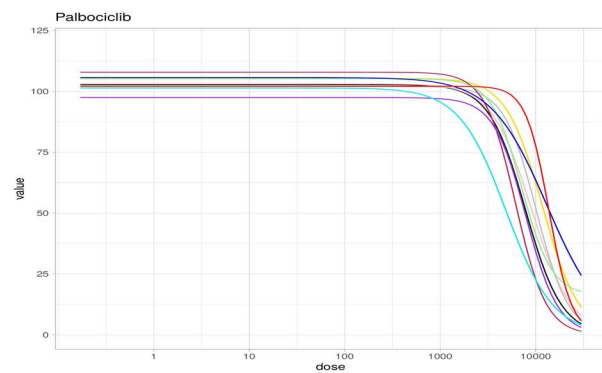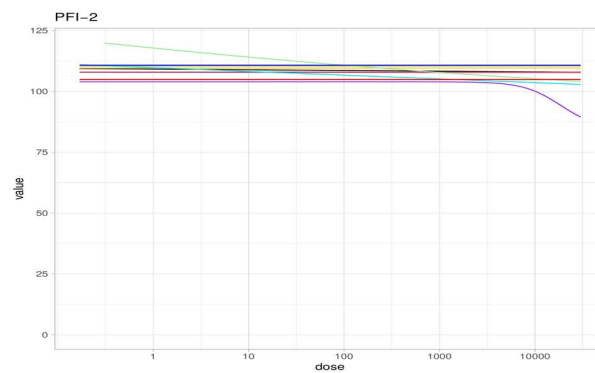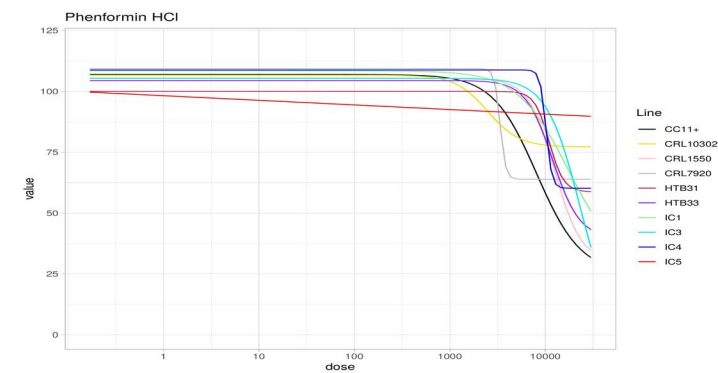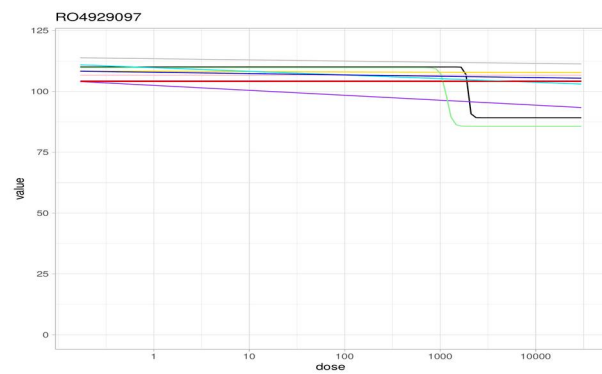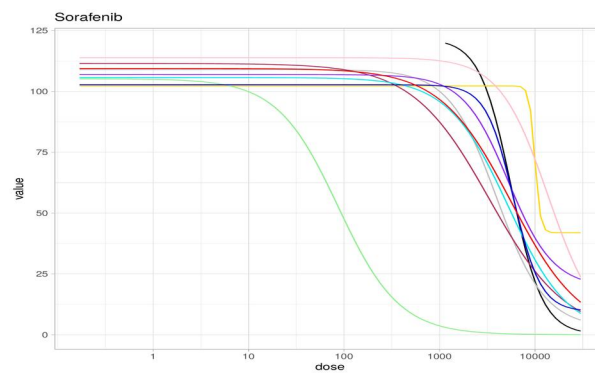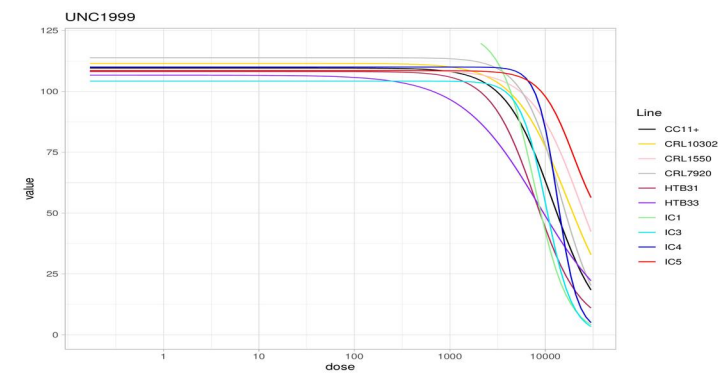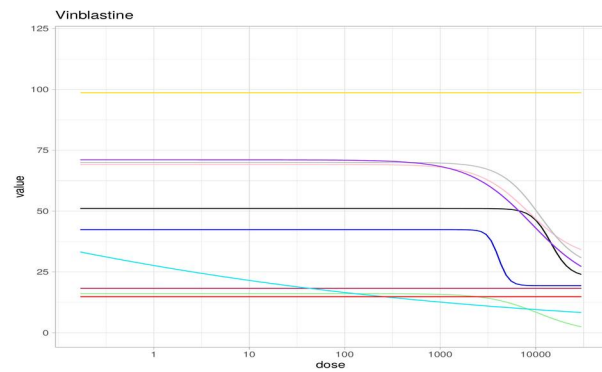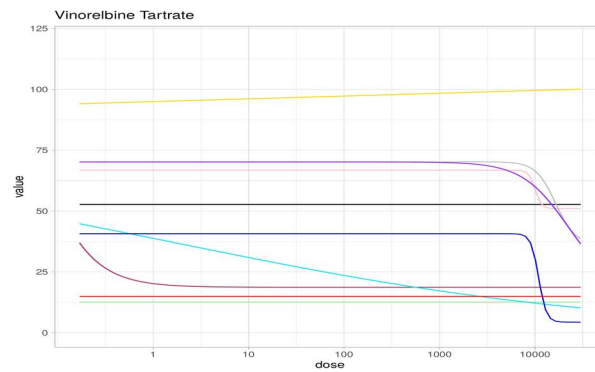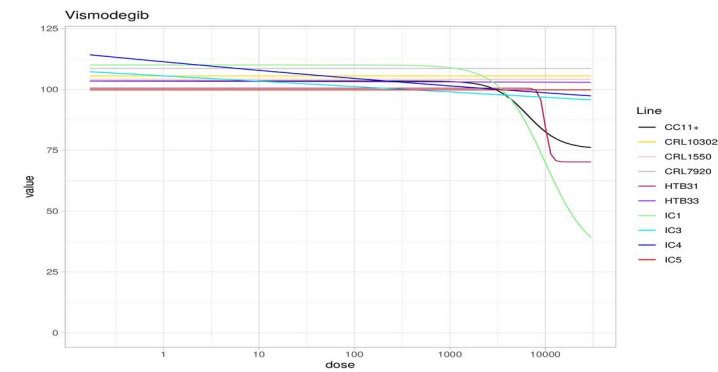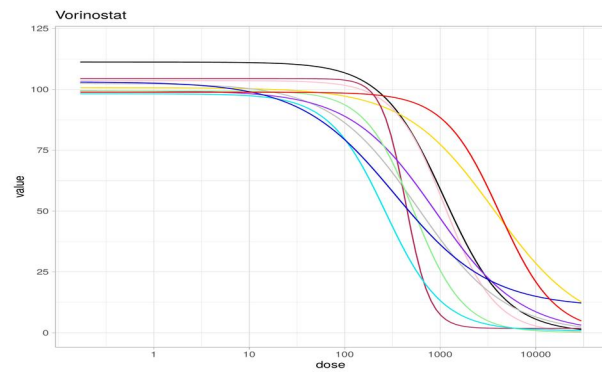

Supplement: Supplementary file 3 — Figure S3: Drug response profiles (IC50) in 10 selected cell lines. IC50, half maximal inhibitory concentration for drugs tested from nM (1) to 10 μM (10000) ranges. (A) Response profiles of APR246, Azacytidine, Botertomib, Carboplatin, CH5132799, Cisplatin, Colchicin, Crizotinib, Dasatinib, Erlotinib, Gefitinib, Gemcitabin. (B) Response profiles of GSK650394, Herceptin, Imatinib, L6, Lapatinib, Merformin, Methotrexate, Mitomycin C, MK‐2206 2HCI, Olaparib, Omipalisib, Paclitaxel. (C) Reponse profiles of Palbociclib, PFI‐2, Phenformin HCI, RO4929097, Sorafenib, UNC1999, Vinblastin, Vinorelbin tartrate, Vismodegib, Vorinostat. [file CNR2-9-e70599-s007.pdf]

## Slide 1
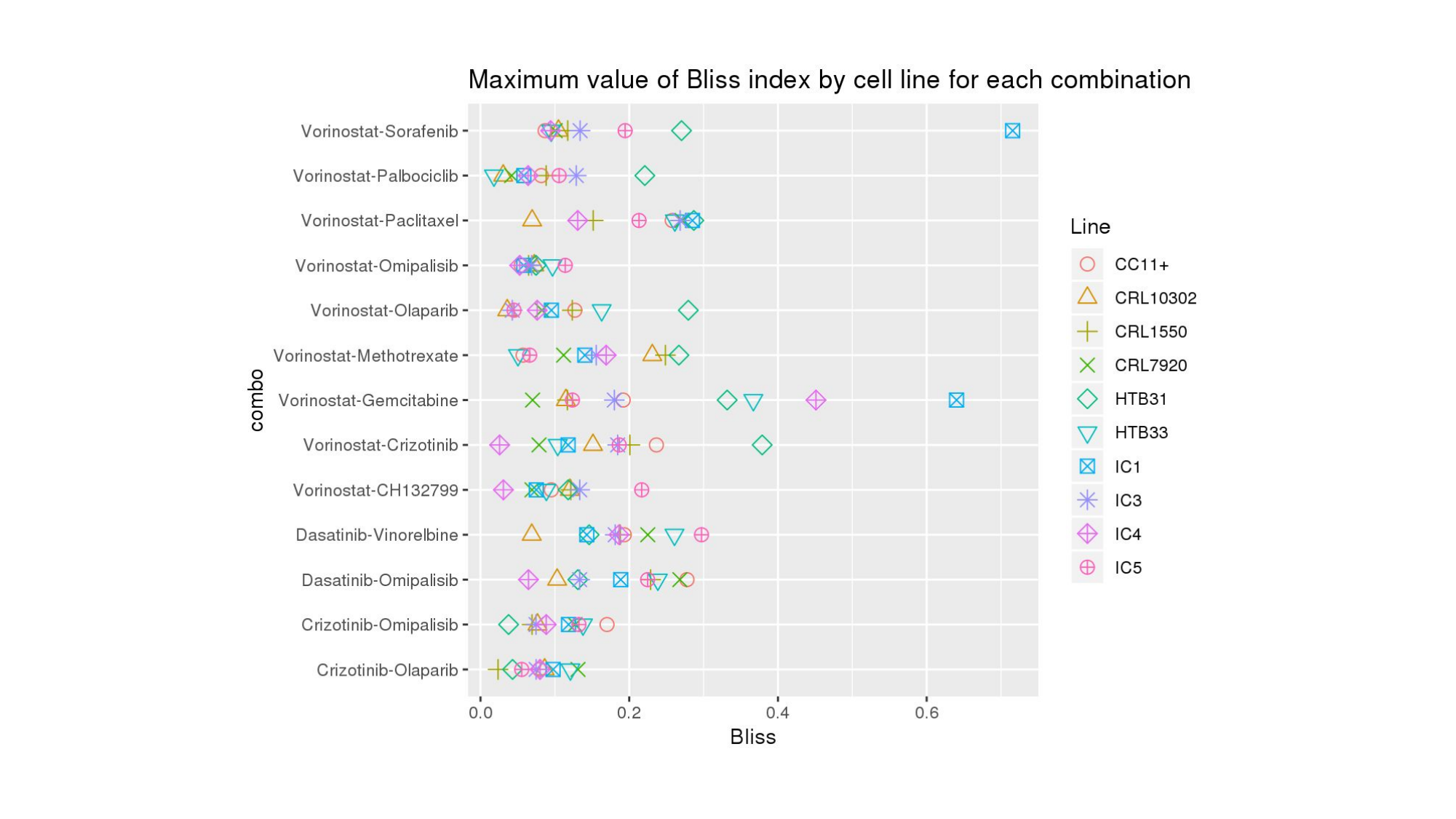

Supplement: Supplementary file 4 — Figure S4: BLISS INDEX summary for each cell line by drug combination. See details under Figure 3. Combo, combination. [file CNR2-9-e70599-s008.pptx]

## Slide 1
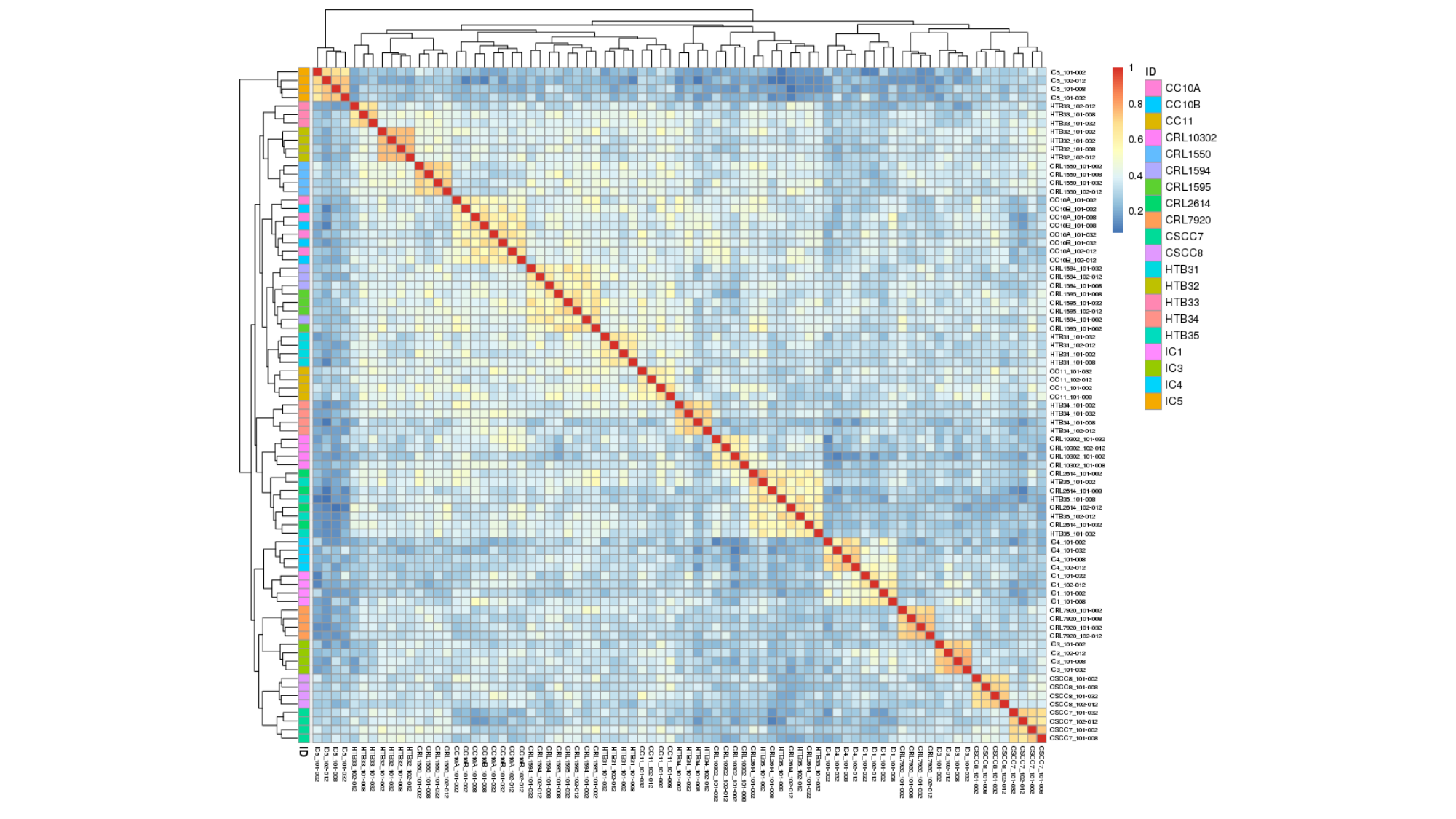

Supplement: Supplementary file 5 — Figure S5: A correlation heatmap (Pearson test); Pearson's correlation coefficient (r) was used to assess the strength and direction of linear relationships between continuous variables, with statistical significance set at p < 0.05. [file CNR2-9-e70599-s004.pptx]
