# Supplementary material for: Unveiling Candidate Markers for Drug Resistance or Synthetic Lethality in Cervical Cancer: Integrative Analysis of Genetic and Pharmacoprofiling
Source: Cancer Rep (Hoboken). 2026 Jun 12;9(6):e70599. doi: 10.1002/cnr2.70599 (PMC13263414; doi:10.1002/cnr2.70599)
Supplement: Supplementary file 6 — Data S1: cnr270599‐sup‐0006‐Supinfo.docx. [file CNR2-9-e70599-s006.docx]

**Supplementary Materials and Methods**

**Cell culture:**

Genomic analysis was based on 20 CLs including, 16 public CLs, described in Kloth et al. [5] and all CL data are recapitulated in **Table 1**. All cell lines with the exception of IC1 to IC5, were obtained from the laboratory headed by Laurence LAFANECHERE (INSERM U1052_CNRS UMR5286). IC3 and CRL10302 cells were cultured in Dulbecco's Modified Eagle Medium (Gibco, Life Technologies), supplemented with 10% fetal bovine serum (Gibco, Life Technologies), 1% Penicillin/Streptomycin (Gibco, Life Technologies); while IC1, IC4, IC5, HTB31, CRL1550, CRL7920 and CC11+ cells were cultured in RPMI 1640 Medium (Gibco, Life Technologies), supplemented with 10% fetal bovine serum (Gibco, Life Technologies), 1% Penicillin/Streptomycin (Gibco, Life Technologies). HTB33 cells were cultured in McCoy 5A Medium (Gibco, Life Technologies), supplemented with 10% fetal bovine serum (Gibco, Life Technologies), 1% Penicillin/Streptomycin (Gibco, Life Technologies) in a 37°C incubator with 5% CO2. At passaging, the cells were washed with PBS and detached with trypsin-EDTA (Gibco Life Technologies) for 5 minutes at 37°C. The screen was performed at same early cell passages (±2) for all replicates, when the cells had been passaged five times, after thawing from liquid nitrogen. To obtain the desired amount of the cells for the screen the cells were counted using T4 Cellometer (Nexcelom). CLs raised at different timepoints from the same patient as well as CLs with low proliferation rates were excluded from pharmacological profiling.

**Screening workflow:**

Drug families investigated included those acting on: (1) DNA repair inhibition: Cisplatin, Carboplatin and Parp inhibition by Olaparib; (2) Epigenetic targeting agents (ETA) through histone deacetylation: Vorinostat, Azacytidine or EZH2 inhibition: UNC1999; (3) receptor tyrosine kinase inhibition: EGFR pathway/VEGFR/PDGFR; (4) nuclear hormone receptor inhibition; (5) inhibition of energy metabolism: biguanides; (6) antimetabolites: 5FU, Methotrexate, Gemcitabine; (7) Microtubule targeting agents (MTAs): Paclitaxel, Colchicine, Vinblastine, Vinorelbine; (8) proteasome degradation; and (9) p53 modulation (APR246).

Cell line densities were empirically determined: 1250 cells per well for IC1 ; 1400 cells per well for HTB33 ; 1500 cells per well for IC3 ; 2000 cells per well for IC4 and IC5 ; 2500 cells per well for CRL1550, CRL7920, CRL10302 and CC11+ and 3000 cells per well for HTB31. Cells were counted with a T4 Cellometer cell counter (Nexcelom) and then seeded in 384-well plates (ViewPlate-384 Black Perkin Elmer, ref. 6007460) in 40µL of media using MultiDrop combi (Thermo Fisher Scientific). Lists of the drugs used: 22 chemical compounds were obtained from Selleckchem Chemicals, 4 compounds were obtained from Sigma Aldrich, 1 from Marc Billaud’s lab (INSERM U1052_CNRS UMR5286), 1 from Biophenics’ lab, 1 from APREA AB, 1 from RT² ‘s lab and 4 from the Institute Curie’s pharmacy (recapitulated in **Table 2**). All compounds were diluted in Dimethyl Sulfoxide (DMSO) and DMSO was added in remaining wells, and represent internal controls. Stock compound plates contain 10mM, excepted for Paclitaxel (3.3mM) and Herceptin (13.7µM). 24 hours after seeding, the dilutions from 10mM to 2mM and from 2mM to 90µM have been done in their respective media. Then, 20µL of compounds were transferred to the cell plates using the MultiChannel Arm™ 384 (MCA 384) (TECAN) to the cells, to a final concentration of 30µM and 0.3% of DMSO (highest concentration). Sequential dilutions 1:3 were done until 0.17 nM. The assay plates were then incubated 48h at 37°C at 5% prior to cell viability assessment.

**Cell viability evaluation:**

CellTiter-Glo 2.0 Assay kit (Promega Inc., Madison, USA) was used immediately after cell seeding to determine the cell viability. The CellTiter-Glo 2.0 reagent was equilibrated to room temperature prior to use. A volume of 25µL per well of CellTiter-Glo 2.0 reagent, was robotically added. The contents were mixed for 2 minutes at 1000 rpm on an orbital shaker (Titramax 100, Dutscher) and plates were further incubated for 10 minutes at room temperature to stabilize luminescent signals. Units of luminescent signal generated by a thermo-stable luciferase are proportional to the amount of ATP present in viable cells. Luminescence was recorded using a CLARIOStar (BMG Labtech).

**Drug response assessment:**

Normalization step: Raw data was cleaned from outlier datapoints. Data from viability were normalized according to the DMSO mean of all the plates of the considered replicate: $\% Viability= \frac{RLU sample value}{mean (RLU DMSO value for the replicate)}\times100$. Each plate (containing data for one combination for one cell line) is normalized by diving the luminescence value of each well by the median of luminescence value of DMSO wells. Therefore, the normalized value can be interpreted as a survival index where a value of 1 indicates no cell death (same luminescence as DMSO wells) and a value of 0 no surviving cells.

Estimation of single drug responses: In a second round of experiments, 10 CL of interest were selected and rescreened at the BioPhenics platform, Institut Curie for their response to 12 targeted or common drugs and for the combination of 13 pairs of drugs at drug dosages reaching from 0 to 10 000 nM, in gradual concentration of 1/3 dilutions. Experiments were carried out in triplicates. Drug exposure time was 48h. The dose response curve for each single drug was estimated by fitting a 4 parameters log-logistic model. Drug response was interpreted as Good, Bad or Intermediate by ranking the cell lines by the following mode parameters (DSS, drug sensitivity score and IC50).

Combined drug analysis: Pharmacological profiles of high interest were repeated for 12 drugs, both on their own and in combination. A Bliss index was calculated after triplicate aggregation, allowing to assess the presence or absence of a synergetic effect of drug combinations in terms of prediction of mortality when combined as compared to the prediction when the drugs were used individually. The Bliss index is a measure of potentiation between two drugs, combined respectively at doses \(i\) and \(j\), it is defined as :\[Bliss_{ij} = E_{i0} * E_{0j} - E_{ij}\] where \(E_{i0}\) and \(E_{0j}\) are the effects of the monotherapies for the two drugs at their respective doses and \(E_{ij}\) the effect of the combination. This Bliss index represents the mortality excess over the independence model for each of the two drugs. For instance, a Bliss index of 0.3 shows 30% of mortality in excess for the combination and a Bliss index of -0.2 shows 20% less mortality for the combination. For each combination, three replicates were aggregated, and median values of the normalized luminescence was retained. The Bliss index was computed for pairs of drugs.

**Whole Exome Sequencing (WES):**

Whole Exome Sequencing of 20 Cervical Cancer Cell Lines available to the RAIDS consortium was based on Illumina paired-end sequencing. Sequencing was performed on the 16 public cell lines, described in Kloth et al. [5] at SeqOmics and on 4 patient derived cell lines, sequenced at Institut Curie not previously referenced. A first quality control report was generated by use of the Ewok (Exome workflow) pipeline; raw reads were aligned on the hg19 reference genome using BWA mem algorithm (v0.7.15). Alignments were filtered out for PCR duplicates, for reads aligning outside of the targeted regions using respectively Picard (v2.6.0) and BEDtools (v2.21.0). Low confidence alignments (mapping quality below 20) were discarded with SAMtools (v0.1.19). Remaining alignments were recalibrated using GATK Base Recalibrator (v4.0.2.1). On average, the total number of reads were 188.259.027 with 8,68 % of duplicates, 65 % were on target at MAPQ20, mean depth was: 148X. Hierarchical clustering based on polymorphisms highlights the right pairing between CRL1594/CRL1595 and CC10A/CC10B but also shows a proximity between cell lines CRL2614 and HTB35 **(Supplementary Figure 2).** HTB31 cell lines had a significantly higher number of frameshift deletions and non-coding variants, consistent with its MSI-High pattern, verified using msi-sensor.

**Reverse Phase Protein Arrays (RPPA):**

20 cell lines had been processed together with the 154 baseline tumour samples and 103 post treatment samples as previously reported [1]. Arrays were labelled with 194 specific antibodies, using an Autostainer Plus (AGILENT). All primary antibodies used in RPPA had been previously tested by Western Blotting to assess their specificity for the protein or phosphoprotein of interest [1,2,4].

**Bioinformatics analysis of genetic alterations potentially linked with drug response.**

In the assessment of genetic alterations potentially linked with drug response, only genes altered in at least 2 cell lines for which the drug response was available and genes which were altered (gain or loss of function) in all good responders or in all bad responders were considered. A wider screen including genetic variants with presently unknown clinical significance **(Supplementary Figure 1)** was subsequently used to detect genetic markers that may be associated with resistance to therapy or with synthetic lethality.
