# Supplementary material for: Unveiling Candidate Markers for Drug Resistance or Synthetic Lethality in Cervical Cancer: Integrative Analysis of Genetic and Pharmacoprofiling
Source: Cancer Rep (Hoboken). 2026 Jun 12;9(6):e70599. doi: 10.1002/cnr2.70599 (PMC13263414; doi:10.1002/cnr2.70599)
Supplement: Supplementary file 9 — Table S3: Microsatellite number of mutations in the 20 cell lines. [file CNR2-9-e70599-s003.docx]

| **Cell Lines** | **Total_Number_of_Sites** | **Number_of_Somatic_Sites** | **%** |
| --- | --- | --- | --- |
| CC10A | 1243 | 34 | 2.74 |
| CC10B | 1240 | 30 | 2.42 |
| CC11 | 1242 | 43 | 3.46 |
| CRL10302 | 1251 | 33 | 2.64 |
| CRL1550 | 1250 | 20 | 1.60 |
| CRL1594 | 1248 | 14 | 1.12 |
| CRL1595 | 1251 | 19 | 1.52 |
| CRL2614 | 1241 | 34 | 2.74 |
| CRL7920 | 1248 | 21 | 1.68 |
| CSCC7 | 1247 | 25 | 2.00 |
| CSCC8 | 1249 | 50 | 4.00 |
| HTB31 | 1251 | 1031 | 82.41 |
| HTB32 | 1249 | 25 | 2.00 |
| HTB33 | 1249 | 24 | 1.92 |
| HTB34 | 1248 | 31 | 2.48 |
| HTB35 | 1236 | 33 | 2.67 |
| IC1 | 1235 | 39 | 3.16 |
| IC3 | 1233 | 34 | 2.76 |
| IC4 | 1238 | 37 | 2.99 |
| IC5 | 1246 | 42 | 3.37 |

**Supplementary Table 3: Microsatellite number of mutations in the 20 cell lines**
